# Supplementary material for: Learning curve and surgical outcome of robotic assisted colorectal surgery with ERAS program
Source: Sci Rep. 2022 Nov 29;12:20566. doi: 10.1038/s41598-022-24665-w (PMC9709162; doi:10.1038/s41598-022-24665-w)
Supplement: Supplementary file 1 — Supplementary Information 1. [file 41598_2022_24665_MOESM1_ESM.zip › LSC Raw data-Ñ[▒K/129/H_ñw╝╨░O▒Kñσ.pdf]

正式摘要(電子簽章：000000221047240)	【讀取】憑證
W52 / W52　☐ 英文標題(注意:無英文姓名-請補病人基本檔!)	

臺中榮民總醫院

出院病歷摘要

轉入號：　　　　轉出號：

[redacted]

(8)流水編號	
(9)入院日期	110/07/08 (2021)
(10)轉科日期	
(11)出院日期	110/07/14 (2021)　住院天數計 6 日

診斷

- 入院：1. Colon polyps, at 10cm and 70cm FAV, s/p colonoscopic biopsy (110/6/21)
  2. Appendicitis, s/p appendectomy in 30+ years ago
  3. HTN
  4. DM
  5. Hemorrhoid
- 出院：1. Colon polyps, at 10cm and 70cm FAV, s/p colonoscopic biopsy (110/6/21), r/o A-colon cancer, s/p laparoscopic RH on 2021/07/09
  2. Appendicitis, s/p appendectomy in 30+ years ago
  3. HTN
  4. DM
  5. Hemorrhoid

主訴

- Positive occult blood test of stool in June, 2021

病史

- This 64-year-old man has past history of:
  1. Appendicitis, s/p appendectomy in 30+ years ago
  2. HTN
  3. DM
  4. Hemorrhoid

  During health check-up in June, 2021, occult blood test of stool was positive. He then came to our hospital, receiving colonoscopy (110/6/21) up to cecum, normal colonic mucosa, one LST around 3-4cm in size at 70cm FAV s/p biopsy as Tubulovillous adenoma; another polyp around 0.3cm in size at 70cm FAV s/p biopsy as Tubular adenoma; one 0.3cm in size polyp at 10cm FAV, s/p hot-snare biopsy+hemoclip*2, pathology reported as Hyperplastic polyp.
  Under the impression of colon polyps, r/o A-colon cancer, the patient was admitted for laparoscopic RH scheduled on 2021/07/09.

體檢發現

-
  - General appearance :
    - easy looking , conscious clear , without cardiopulmonary distress
    - Vital signs自動帶入date 體溫(BT) 脈搏(P) 呼吸(R) 血壓(BP) 身高(BH) 體重(BW) BMI
      20210708 36.6℃ 96次/分 18次/分 128/62mmHg 165.2cm 71kg 26.0
    - Vital signs: B.P. 128/62 mmHg , P.R. 96 /min , R.R. 18 /min , B.T. 36.6 ℃
  - HEENT :
    - Eyes: no anemic conjunctiva, no icteric sclera, normal EOM
    - ENT: no otorrhea, no rhinorrhea, no erythematous mucosa, no injected tonsil, no deformity
  - Neck: supple, freely movable, trachea midline,no deformity, no engorged jugular vein , no palpable thyroid or lymph node enlargement
  - Chest:
    - Inspection: Grossly normal, symmetric and fully expansion, no accessory muscle used, no operation scar, no spider angiomata, no gynecomastia
    - Palpation: no palpable mass or nodule, no subcutaneous emphysema, no tenderness

-
    - Ausculation: clear breathing sound
- Heart:
    - Palpation: no thrill, no heave
    - Percussion: normal heart size
    - Ausculation: regular heart beat, normal S1, S2, no gallop, no murmur
- Extremities: No deformity, full ranged of motion without limitation, no clubbing finger or nail cyanosis, no pitting edema, no ulcer or pigmentation, no obvious skin lesion, no varicose vein, no bone, joint or muscle tenderness

CRS CONDITION

- Karnofsky performance status: (100) Normal, no complaints, no evidence of disease
- PE of Abdomen:
- CEA: 1.93 ng/ml
- Imaging Studies:
- Colonoscope report: Colonoscopy (110/6/21) up to cecum, normal colonic mucosa, one LST around 3-4cm in size at 70cm FAV s/p biopsy as Tubulovillous adenoma; another polyp around 0.3cm in size at 70cm FAV s/p biopsy as Tubular adenoma; one 0.3cm in size polyp at 10cm FAV, s/p hot-snare biopsy+hemoclip*2, pathology reported as Hyperplastic polyp
- Figures:
    1. colon & rectum

手術日期及方法

- 手術日期及方法20210709 1. Laparoscopic right hemicolectomy with side to side anastomosis (OpenGIA 80-3.8 X2 were applied)
  2. Ileo-colic artery, right colic artery and right branch of middle colic artery were all ligated via Hemo-clips.
  3. Take down of hepatic flexure : (+)
  4. Cut margin: proximal (ileum) 6 cm, (colon) 5 cm, distal 8 cm.
  5. Repair of mesentery defect : Nil.
  6. No drainage tube was placed.

住院治療經過

- After operation with laparoscopic RH on 2021/07/09, the patient was under post-operative care and pain control. Self-urination was smoothly after removal and re-insertion of Foley's catheter on 7/10, then removal on 7/12. The patient tried water since 7/10, then tolerated Bland No.I diet on 7/11, No.II diet on 7/12, and No.III diet since 7/13, with nausea sensation on 7/12, without vomting, abdominal pain or discomfort. The wound was clean and dry, without infectious signs. Under relative stable condition, the patient was discharge with OPD follow up.

合併症

一般檢查

- 累積報告：

DATE	TIME	ALB	TP	BIL.T	BIL.D	ALKP	AST	ALT	LDH	AMY	LIPASE	NH3	Ferritin	UA
1100709	1221	4.1		1.14			40	63						

DATE	TIME	WBC	RBC	HGB	HCT	MCV	PLT	BLST	NEUT	BAND	SEG	LYM	MON	EOS	BAS
1100709	0729			13.1	42.6		265								
1100709	1221	15370		13.2			249		85.1			11.1	3.3	0.3	0.2

DATE	TIME	NA	K	CL	BUN	CREAT	eGFR	CA	Mg	P	CRP	ESR	PCT	D-Dimer	LACTATE
1100709	1221	139	4.2		11	0.98									

DATE	TIME	GLUCOSE	HbA1C	CHOL	TG	HDL	LDL	KETONE	CK	CKMB	TRO-T	PT-P	APTT-P	NT-pro BNP
1100709	0729											10.7		

特殊檢查

病理報告

- 活體組織切片病理報告　手術日期：**110/07/09**　病理號：**S1021053**

  診斷醫師:楊啟順 報告日期:110/07/15
  Pathologic diagnosis:
  Colon, and terminal ileum, right hemicolectomy --- Tubulovillous adenoma with low grade dysplasia. The ileocecal valve, bilateral cut ends and mesocolic lymph nodes (0/19) are free of tumor.

- Ancillary study for diagnosis:
  Deep cut done on section D and E.
  Gross description:
  The specimen consists of a segment of colon, 17 cm in length, and 8.5 cm in proximal circumference, 10.5 cm in distal cut end circumference, and a segment of terminal ileum, 8.5 cm in length, and 4 cm in cut end circumference. No appendix is noted. A sessile polyp, 2x1 cm, is noted 8 cm to the nearest cut end. The ileocecal valve, and both cut ends are free of tumor. Representative part for section:
  A) proximal cut end B) distal cut end C) ileocecal valve D-E) polyp
  F-H) lymph nodes.
  #T-59420_2 #M-82630_2 0 1105 000000
  #T-C4560_2 #M-09183_2 0 1105 000000
  #T-59200_2 #M-09183_2 0 1105 000000

其它

出院時情況

- 3.改門診治療

出院指示

藥名	劑量	劑量單位	用法	頻次	數量	數量單位	現況	天數	單位
Bokey cap 100mg	1	CAP	PO	QD	13	CAP	TH	13	天
Glucomet-5/500 tab	2	TAB	PO	BID	52	TAB	TH	13	天
(口)Takepron OD tab 30mg	1	TAB	PO	QDAC	13	TAB	TH	13	天
Trajenta tab 5mg	1	TAB	PO	QD	13	TAB	TH	13	天
Mag. Oxide tab	2	TAB	PO	TID	78	TAB	TH	13	天
Trazodone tab 50mg	1	TAB	PO	HS	13	TAB	TH	13	天
Ultracet tab	1	TAB	PO	BID&HS	39	TAB	TH	13	天
Amtrel tab 5/10mg	2	TAB	PO	QD	26	TAB	TH	13	天
100mg Acarbose tab	1	TAB	PO	BIDCC	26	TAB	TH	13	天
Through tab	1	TAB	PO	QN	13	TAB	TH	13	天
OCAS Harnalidge 0.4 mg	1	TAB	PO	QDAC	13	TAB	TH	13	天
Livalo tab 2mg	1	TAB	PO	QN	13	TAB	TH	13	天
20g MY comb Cream		TUB	TOP	BID	0	TUB	TH	1	瓶

出院計劃

- 門診日期
  - date on 2021/07/20

主治醫師_2062F 蔣鋒帆 / 住院醫師_2345B 陳映晴

正式摘要(電子簽章：000000221047240)

W52 / W52

doctorRank:B
